# Elevated Serum Gamma-Glutamyltransferase Is a Strong Marker of Insulin Resistance in Obese Children

**DOI:** 10.1155/2013/578693

**Published:** 2013-03-12

**Authors:** Seon Yeong Lee, Eunju Sung, Yoosoo Chang

**Affiliations:** ^1^Department of Family Medicine, Inje University, College of Medicine, Sanggyepaik Hospital, Nowon-Gu, Seoul 139-707, Republic of Korea; ^2^Department of Family Medicine, Kangbuk Samsung Hospital, Sungkyunkwan University, School of Medicine, 108 Pyongdong, Jongro-Gu, Seoul 110-746, Republic of Korea; ^3^Department of Occupational Medicine, Kangbuk Samsung Hospital, Sungkyunkwan University, School of Medicine, 108 Pyongdong, Jongro-Gu, Seoul 110-746, Republic of Korea; ^4^Health Screening Center, Kangbuk Samsung Hospital, Sungkyunkwan University, School of Medicine, Seoul 110-746, Republic of Korea

## Abstract

Elevated levels of serum gamma-glutamyltransferase (GGT) levels have been found to predict the development of type 2 diabetes in adults. The role of GGT in insulin resistance (IR) among children is largely unknown. We investigated whether GGT among hepatic enzymes is independently associated with IR in obese Korean children. A total of 1308 overweight (above the 85th BMI percentile of Korean reference) boys (*n* = 822) and girls (*n* = 486), aged 9–15 years, were studied. Measures acquired included weight, height, percent body fat (BF%), waist circumference, blood pressure, blood glucose and insulin, C-reactive protein, total cholesterol, triglycerides, HDL-Cholesterol, GGT, aspartate aminotransferase (AST), and alanine aminotransferase (ALT). IR was calculated using the homeostasis model assessment (HOMA-IR). Serum GGT and ALT, but not AST, were positively correlated with HOMA-IR in boys (*r* = 0.222 for GGT; *P* < 0.05, *r* = 0.188 for ALT; *P* < 0.05) and girls (*r* = 0.292 for GGT; *P* < 0.05, *r* = 0.258 for ALT; *P* < 0.05). In multiple regression analysis for HOMA-IR as dependent variable, GGT (*β* = 0.068; *P* = 0.053 in boys, *β* = 0.145; *P* = 0.002 in girls) and ALT (*β* = 0.074; *P* = 0.034 in boys, *β* = 0.130; *P* = 0.005 in girls) emerged as determinants of HOMA-IR after adjusting age, BMI, tanner stage, and triglycerides. Serum GGT level is a strong marker of IR in obese Korean children.

## 1. Introduction

Obesity is a risk factor for type 2 diabetes mellitus, and its prevalence is increasing worldwide. Moreover, an alarming increase in the incidence of type 2 diabetes has now been reported in very young children [1]. Insulin resistance (IR), the most common feature of childhood obesity, is a key risk factor for development of type 2 diabetes [2]. Serum activities of hepatic enzymes have been associated with obesity in adults [3] and adolescents [4], and relationships between these markers and IR or type 2 diabetes, independently of adiposity, have been shown in several studies [5–8]. In adult population, recent studies indicate that alanine aminotransferase (ALT) [8, 9] and gamma-glutamyltransferase (GGT) are independent predictors of type 2 diabetes [6, 7, 10]. Indeed, ALT is a glucogenic enzyme and is regarded as a surrogate marker of hepatic IR, nonalcoholic fatty liver disease (NAFLD) [11, 12]. Increased ALT has been demonstrated to be an indicator of impaired insulin signaling [13]. On the other hand, serum GGT is one of the hepatobiliary enzymes and is synthesized in epithelial cells of the intrahepatic duct. Serum GGT traditionally has been used as a marker of alcohol-induced liver disease [14]. Recently, GGT has been regarded as a clinical marker for free-radical formation and proinflammation [15]. GGT-related pathomechanism is that GGT enhances the availability of cysteine to promote intracellular glutathione, the principle thiol antioxidant in humans, and resynthesis, thereby counteracting oxidant stress [16]. The relationships of liver enzyme with IR in children are less likely to be affected by alcohol, comorbidities, and medicine, which might influence the liver function, than in adults. However, studies that clarify whether the liver plays an early role in the natural history of the disease in children with no or minimal cumulative exposure to unhealthy behaviors are rare. The aim of this cross-sectional study is to evaluate whether an independent relationship exists between insulin resistance and serum hepatic enzyme activities in obese Korean children. 

## 2. Materials and Methods

### 2.1. Subjects

#### 2.1.1. Study of Seoul Obese School Children (SSOS)

This study was designed to identify the prevalence of risk factors and relationship between risk factors and metabolic profile in obese school children in Seoul. All primary and middle schools in Seoul were invited to join the study. Among them, 25 schools (15 of primary schools and 10 of middle schools) were selected by order of their responses. The target study subjects were overweight and obese students, over 85th percentile of age, and gender-specific Korean BMI references [17] in annual school examination. Anthropometric measurement, blood test, and questionnaire were performed to children whose parents and themselves agreed to join the study. A team consisting of 5 medical doctors and 3 nurses and 3 assistants were sent to the school for the measurements. After all the examination, one doctor consulted students who are regarded as having serious medical or behavioral difficulties. This study (The Study of Seoul Obese School Children) was performed as a part of children's obesity-related disease management program conducted by Seoul School Health Promotion Center, Study Group of Childhood Obesity, and Obesity Clinic in Kangbuk Samsung Hospital and Inje University Sanggye Paik Hospital. This study was approved by Institutional Review Board of Kangbuk Samsung Hospital and Inje university Sanggye Paik Hospital. Total of 1716 children (1105 boys, 611 girls, aged 9–15 years old) were enrolled. 

For this study, subjects were 1308 (822 boys and 486 girls) after excluding children with established diabetes, recent infection, suspected endocrinological disease, positive for hepatitis B viral markers, and not fasted for at least 9 hours.

### 2.2. Study Procedures

#### 2.2.1. Anthropometric and Blood Pressure Measurements and Physical Examinations

Height (cm) was measured to the last complete millimeter, with the subjects barefoot, using a wall-mounted stadiometer. Weight (kg) was measured to 100 g on a calibrated digital electric scale. Body mass index (BMI) was calculated as weight/height^2^ (kg/m^2^). BMI percentile was calculated using Korean Children BMI Chart according to age and gender [17]. Waist circumference was measured by a trained person, to the nearest 0.1 cm at the midpoint between the bottom of the rib cage and the top of the iliac crest with the subjects standing, their weight equally distributed on both feet, their arms at their sides, and head facing straight forward. Body fat percentage (BF%) was measured by bioelectrical impedance analysis (Inbody 720, Biospace Inc, Seoul, Korea) [18]. Blood pressure (mmHg) was measured in a seated position after relaxation for 5 minutes using an automated oscillometric blood pressure recorder (Dinamap procure 100, GE medical system) [19]. Arm circumference was measured on the appropriate cuff size. Seated blood pressure was taken twice in succession (at 1-minute interval) from both arms, and an additional measurement was performed on the right arm if BP difference was more than 10 mmHg. Their means of the measurements were used in all analyses. Tanner stage was assessed by self-administered questionnaire.

#### 2.2.2. Biochemical Measurements

 After midnight fast, blood samples were drawn from an antecubital vein. Hepatitis B surface antigen (HBs Ag) was measured using a commercially available immunoradiometric assay (Riakey, Korea). Total cholesterol, glucose, HDL- (high-density-lipoprotein-) cholesterol, triglycerides, ALT, AST, and GGT were measured (Hitachi 747 automatic analyzer, Hitachi, Japan) and fasting serum insulin by immunoradiometric assay (Biosource, Nivelles, Belgium). Serum C-reactive protein (CRP) was analyzed using immunonephelometry assay (Dade Behring, Marburg, Germany). The homeostasis model for the calculation of an insulin resistance (HOMA-IR) index was used where HOMA-IR = fasting glucose (mmol/L) × fasting insulin (*μ*U/mL)/22.5 [20].

### 2.3. Statistical Analysis

The data are presented as mean ± standard deviation (SD) or the absolute number (percentages). Differences between boys and girls were assessed using *t*-tests. Correlation analyses were conducted between GGT, ALT, and AST and anthropometric, metabolic, and IR variables, after adjustment for age in boys and girls, separately. The hypothesis that GGT is a significant marker of HOMA-IR was tested using multiple linear regression analysis where HOMA-IR was the dependent variable and serum GGT or ALT, age, tanner stage, BMI, and triglycerides were independent variables. The level of significance for statistical tests was *P* < 0.05. All statistical analyses were performed using STATA version 11.0 (StataCorp LP, College Station, TX, USA).

## 3. Results

### 3.1. Baseline Characteristics

The descriptive characteristics for this study subjects are presented for boys and girls separately in Table 1. 

The prevalence of obese children (BMI > 95th percentile) is more than 70% (70.2% in boys, 78.8% in girls). There were no gender differences in anthropometric variables except body fat percent. Among the metabolic variables, fasting glucose and insulin, HOMA-IR, and liver enzymes (GGT, ALT, AST) showed gender differences. Fasting glucose levels in boys are higher than those in girls, but fasting insulin levels in girls are higher than those in boys. The levels of liver enzymes in boys are higher than in girls. And there was a gender difference in tanner stage: the girls had higher tanner stage than boys (*P* < 0.001). 

### 3.2. Relationship between Insulin Resistance and Liver Enzymes, Metabolic Variables

Tables 2 and 3 showed that serum activities of all three liver enzymes were positively correlated with BMI, waist circumference, and BF% after adjustment for age in boys and girls, respectively. 

Serum GGT and ALT, but not AST, were positively correlated with HOMA-IR in boys (*r* = 0.222 for GGT; *P* < 0.05, *r* = 0.188 for ALT; *P* < 0.05) and girls (*r* = 0.292 for GGT; *P* < 0.05, *r* = 0.258 for ALT; *P* < 0.05). Waist and triglycerides was the most correlated factor with HOMA-IR in boys (*r* = 0.275 for waist; *P* < 0.05, *r* = 0.275 for triglycerides; *P* < 0.05). BMI and GGT presented the highest correlation with HOMA-IR in girls (*r* = 0.347 for BMI; *P* < 0.05, *r* = 0.292 for GGT; *P* < 0.05).

All liver enzymes were positively correlated with total cholesterol and triglycerides and negatively correlated with HDL-cholesterol in boys and girls, respectively. In boys, serum GGT, neither AST nor ALT, was positively correlated with CRP as an inflammation marker. On the other hand, in girls, serum GGT, AST, and ALT were positively correlated with CRP. GGT was correlated with HOMA-IR and CRP more than ALT. In multiple regression analysis for HOMA-IR as dependent variable, GGT (*β* = 0.068; *P* = 0.053 in boys, *β* = 0.145; *P* = 0.002 in girls) and ALT (*β* = 0.074; *P* = 0.034 in boys, *β* = 0.130; *P* = 0.005 in girls) emerged as determinants of HOMA-IR after adjusting age, BMI, tanner stage, and triglycerides (Tables 4 and 5).

## 4. Discussion

In overweight and obese Korean children, we found a significant association serum GGT activity with IR. Although previous adult studies [7] including Korean people have shown similar independent relationships between GGT and insulin resistance, the relationship between GGT and insulin resistance in children has not previously been described except in small population of Pima Indian [21]. A study of 44 Pima Indian children showed GGT, neither AST nor ALT, was a significant determinant of HOMA-IR independently of age, gender, weight, body mass index, or percent body fat [21]. However, The Pima Indians are well-known high risk population for diabetes mellitus; for example, the Pima Indians of Arizona have the highest reported prevalence of diabetes of any population in the world [22, 23]. Therefore, it may not be generalized to other population with average risk for diabetes. In Korean obese children, both GGT and ALT were significant determinants of HOMA-IR independently of age, tanner stage, and BMI in both genders. 

Pubertal development is known to affect insulin status in children, but it was not significant in our obese children [2, 24]. It seems that obesity effect on IR is stronger than pubertal stage in obese Korean children. Further investigations need to clarify the association between obesity and puberty effect to IR. 

The mechanism of the relationship between insulin resistance and GGT has not been clarified. GGT is a cell-surface protein contributing to the extracellular catabolism of glutathione (GSH) [25]. This enzyme is produced in many tissues, but most GGT in serum is derived from the liver [24]. In the serum, GGT is carried primarily with lipoproteins and albumin [26]. Serum levels of GGT are determined by several factors: alcohol intake, body fat content, plasma lipid/lipoproteins, glucose levels, and various medications [25, 27]. Children are better a subject to clarify the role of liver enzyme in IR as they have less alcohol and medication. Increases in GGT activity can be a response to oxidative stress, facilitating increased transport of GSH precursors into cells. In addition, GGT is leaked into the serum possibly as a result of normal cell turnover and cellular stresses [15]. Lee and Jacobs Jr. [28] reported that in the general American population, the serum level of GGT, rather than that of ALT, was more closely associated with the plasma level of CRP, and they speculated that increased oxidative stress related to elevated serum GGT might contribute to this relationship. Our study results showed that serum GGT activity was positively correlated with CRP as an inflammation marker in children. The etiological role of the liver in later development of type 2 diabetes is debatable. Animal models support a role of hepatic insulin resistance leading to severe glucose intolerance [29, 30]. Furthermore, NAFLD, an emerging obesity-related liver disease in children, is a condition associated with elevated concentrations of serum GGT [31]. Insulin resistance is the main pathogenic factor in the etiology of NAFLD in adults [32] and in children [33]. In another Korean adult study [34], serum GGT was closely related with IR and might be more associated with dyslipidemia and abnormal glucose tolerance regardless of the presence of NAFLD, suggesting that increased serum GGT might reflect more the hepatic insulin resistance. The Mexico City Diabetes Study [10], found raised GGT to be associated with the features of metabolic syndrome and to be an independent predictor of diabetes and concluded that this association may reflect both hepatic steatosis and enhanced oxidative stress. 

In our results, obesity and triglycerides were the major determinants of HOMA-IR in boys. These results are compatible with the result of Sinaiko et al., which showed heavy-insulin resistant adolescents had the highest triglycerides level [35].

Obesity and GGT were the major determinants of HOMA-IR in girls. The effect of triglycerides and GGT on IR was different by gender. Steinberger et al. originally reported that the degree of IR, as measured by the hyperinsulinemic euglycemic clamp, explained a significant portion of the variance in the levels of triglycerides, LDL cholesterol, and HDL-cholesterol in obese adolescents [36]. The gender difference of the association of GGT, TG, and IR was first described in our study in children population, as far as we know. The mechanism of gender difference also needs to further investigated. 

The present study has some limitations. The current findings must be interpreted with caution because of the cross-sectional study design. And our subjects are overweight/obese children, certainly not representative of the general children population. We used HOMA-IR as an index of insulin resistance, instead of euglycemic hyperinsulinemic clamp. However, HOMA-IR is more practical and less invasive than the euglycemic clamp and used in many studies in children HOMA-IR as well as indices derived from the oral glucose tolerance test were well correlated with insulin sensitivity measured by the euglycemic hyperinsulinemic clamp technique.

In summary, in overweight and obese Korean children at high risk for the development of type 2 diabetes, serum GGT activity showed a significant relationship with insulin resistance using HOMA-IR. Further investigation of the mechanisms underlying these associations in children is warranted through a prospective study.

## Figures and Tables

**Table 1:** Basic characteristics of the subjects (total no. = 1308).

	Boys (*N* = 822)	Girls (*N* = 486)	*P* value*
Age (years)	11.9 ± 1.7	12.0 ± 1.8	0.330
BMI (kg/m^2^)	26.6 ± 2.9	26.2 ± 3.3	0.072
BMI percentile, no. (%)			0.003
85–89	55 (6.7)	25 (5.1)	
90–94	191 (23.2)	79 (16.3)	
>95	576 (70.1)	382 (78.6)	
BF%	36.4 ± 5.6	38.0 ± 5.3	<0.001
Waist circumference (cm)	88.4 ± 8.3	88.0 ± 8.9	0.436
Systolic blood pressure (mmHg)	116 ± 14	114 ± 13	0.002
Diastolic blood pressure (mmHg)	64 ± 7	65 ± 7	0.090
Fasting glucose (mg/dL)	87.0 ± 6.9	85.4 ± 7.8	<0.001
Fasting insulin (*µ*U/mL)	17.9 ± 11.7	19.9 ± 12.8	0.004
HOMA-IR	3.9 ± 2.7	4.3 ± 2.9	0.025
CRP (mg/dL)	0.2 ± 0.3	0.2 ± 0.4	0.918
Total cholesterol (mg/dL)	176.9 ± 31.5	175.6 ± 29.8	0.479
Triglycerides (mg/dL)	123.8 ± 62.5	128.9 ± 63.4	0.155
HDL-cholesterol (mg/dL)	54.2 ± 12.4	53.0 ± 12.1	0.089
AST (U/L)	34.0 ± 18.1	26.4 ± 10.0	<0.001
ALT (U/L)	47.6 ± 47.5	30.9 ± 20.5	<0.001
GGT (U/L)	28.1 ± 13.8	22.4 ± 8.0	<0.001
Tanner stage, no. (%)			<0.001
1	339 (41.75)	15 (3.13)	
2	196 (24.14)	166 (34.58)	
3	225 (27.71)	199 (41.46)	
4	52 (6.40)	93 (19.38)	
5		7 (1.46)	

Data are presented as mean ± SD except BMI percentile and tanner stage. *by Student's *t*-test.

BMI: body mass index; BF%: body fat percent; CRP: C-reactive protein; HDL: high density lipoprotein; AST: aspartate transaminase; ALT: alanine transaminase; GGT: gamma-glutamyltransferase; HOMA-IR: homeostasis model assessment of insulin resistance.

**Table 2:** Partial correlation of serum GGT, ALT, and AST with other variables and anthropometric variables in boys.

	GGT	ALT	AST	HOMA-IR
BMI	0.3143*	0.3266*	0.1846*	0.2642*
Waist circumference	0.2395*	0.3230*	0.1851*	0.2749*
BF%	0.1584*	0.1192*	0.1278*	0.0092
Total cholesterol	0.3391*	0.2818*	0.2892*	0.0823
Triglycerides	0.2691*	0.2426*	0.2229*	0.2746*
HDL-cholesterol	−0.1342*	−0.1635*	−0.1222*	−0.0717
Fasting glucose	−0.0393	−0.0817	−0.1289*	0.4464*
Fasting insulin	0.2437*	0.2183*	0.1088*	0.9879*
HOMA-IR	0.2224*	0.1875*	0.0814	—
CRP	0.1362*	0.0950	0.0783	−0.0390
Systolic blood pressure	0.2167*	0.2189*	0.1281*	0.1578*
Diastolic blood pressure	0.2366*	0.1961*	0.1147*	0.0976*
GGT	—	0.7890*	0.6290*	0.2224*

*P value < 0.05 by Spearman's partial correlation adjusted for age. GGT, ALT, AST, Fasting insulin, HOMA-IR, and CRP were log-transformed.

BMI: body mass index; BF%: body fat percent; CRP: C-reactive protein; HDL: high density lipoprotein; AST: aspartate transaminase; ALT: alanine transaminase; GGT: gamma-glutamyltransferase; HOMA-IR: homeostasis model assessment of insulin resistance.

**Table 3:** Partial correlation of serum GGT, ALT, and AST with other variables and anthropometric variables in girls.

	GGT	ALT	AST	HOMA-IR
BMI	0.3030*	0.3395*	0.1559*	0.3471*
Waist circumference	0.2814*	0.3125*	0.1675*	0.3225*
BF%	0.1920*	0.1957*	0.1251*	0.1378*
Total cholesterol	0.3001*	0.1989*	0.2580*	0.0755
Triglycerides	0.3312*	0.2576*	0.2723*	0.1154*
HDL-cholesterol	−0.1256*	−0.1324*	−0.0664	−0.0345
Fasting glucose	0.1968*	0.1009*	0.0806	0.3393*
Fasting insulin	0.2750*	0.2541*	0.0804	0.9876*
HOMA-IR	0.2915*	0.2577*	0.0893	—
CRP	0.3392*	0.2118*	0.2153*	0.1326*
Systolic blood pressure	0.1870*	0.1954*	0.1062*	0.1563*
Diastolic blood pressure	0.1550*	0.1122*	0.0439	0.1688*
GGT	—	0.7432*	0.6642*	0.2915*

*P value < 0.05 by Spearman's partial correlation adjusted for age. GGT, ALT, AST, Fasting insulin, HOMA-IR, and CRP were log-transformed.

BMI: body mass index; BF%, body fat percent; CRP: C-reactive protein; HDL: high density lipoprotein; AST: aspartate transaminase; ALT: alanine transaminase; GGT: gamma-glutamyltransferase; HOMA-IR: homeostasis model assessment of insulin resistance.

**Table 4:** Multiple linear regression analysis in boys.

Dependent variable (log HOMA-IR)	Independent variables	B	SE	*β*	*P* value
*R* ^2^ = 0.128	Age	−0.021	0.016	−0.067	0.186
*R* ^2^_adjusted_ = 0.122	Tanner stage	0.015	0.026	0.028	0.566
	BMI	0.045	0.007	0.253	<0.001
	GGT	0.003	0.001	0.068	0.053
	Triglycerides	0.002	0.0003	0.188	<0.001

*R* ^2^ = 0.128	Age	−0.019	0.016	−0.061	0.229
*R* ^2^_adjusted_ = 0.123	Tanner stage	0.016	0.026	0.030	0.539
	BMI	0.045	0.007	0.249	<0.001
	ALT	0.001	0.0004	0.074	0.034
	Triglycerides	0.002	0.0003	0.191	<0.001

Dependent variables is log-transformed HOMA-IR.

ALT: alanine transaminase; GGT: gamma-glutamyltransferase; HOMA-IR: homeostasis model assessment of insulin resistance.

**Table 5:** Multiple linear regression analysis in girls.

Dependent variable (log HOMA-IR)	Independent variables	B	SE	*β*	*P* value
*R* ^2^ = 0.172	Age	−0.067	0.019	−0.210	0.001
*R* ^2^_adjusted_ = 0.163	Tanner stage	0.020	0.040	0.029	0.612
	BMI	0.067	0.009	0.381	<0.001
	GGT	0.011	0.003	0.145	0.002
	Triglycerides	0.001	0.0004	0.075	0.090

*R* ^2^ = 0.168	Age	−0.062	0.020	−0.195	0.002
*R* ^2^_adjusted_ = 0.159	Tanner stage	0.019	0.040	0.027	0.641
	BMI	0.067	0.009	0.378	<0.001
	ALT	0.004	0.001	0.130	0.005
	Triglycerides	0.001	0.0004	0.088	0.044

Dependent variables is log-transformed HOMA-IR.

ALT: alanine transaminase, GGT: gamma-glutamyltransferase, HOMA-IR: homeostasis model assessment of insulin resistance.

## References

[1] Fagot CA, Narayan K (2001). Type 2 diabetes in children. *British Medical Journal*.

[2] Levy-Marchal C, Arslanian S, Cutfield W (2010). Insulin resistance in children: consensus, perspective, and future directions. *Journal of Clinical Endocrinology and Metabolism*.

[3] Clark JM, Brancati FL, Diehl AM (2003). The prevalence and etiology of elevated aminotransferase levels in the United States. *American Journal of Gastroenterology*.

[4] Strauss RS, Barlow SE, Dietz WH (2000). Prevalence of abnormal serum aminotransferase values in overweight and obese adolescents. *Journal of Pediatrics*.

[5] Rantala AO, Lilja M, Kauma H, Savolainen MJ, Reunanen A, Kesäniemi YA (2000). Gamma-glutamyl transpeptidase and the metabolic syndrome. *Journal of Internal Medicine*.

[6] Perry IJ, Wannamethee SG, Shaper AG (1998). Prospective study of serum *γ*-glutamyltransferase and risk of NIDDM. *Diabetes Care*.

[7] Lee DH, Jacobs DR, Gross M (2003). *γ*-glutamyltransferase is a predictor of incident diabetes and hypertension: the Coronary Artery Risk Development in Young Adults (CARDIA) study. *Clinical Chemistry*.

[8] Vozarova B, Stefan N, Lindsay RS (2001). High alanine aminotransferase is associated with decreased hepatic insulin sensitivity and predicts the development of type 2 diabetes. *Diabetes*.

[9] Hanley AJG, Williams K, Festa A (2004). Elevations in markers of liver injury and risk of type 2 diabetes: the insulin resistance atherosclerosis study. *Diabetes*.

[10] Nannipieri M, Gonzales C, Baldi S (2005). Liver enzymes, the metabolic syndrome, and incident diabetes: the Mexico City diabetes study. *Diabetes Care*.

[11] Schindhelm RK, Diamant M, Dekker JM, Tushuizen ME, Teerlink T, Heine RJ (2006). Alanine aminotransferase as a marker of non-alcoholic fatty liver disease in relation to type 2 diabetes mellitus and cardiovascular disease. *Diabetes/Metabolism Research and Reviews*.

[12] Chang Y, Ryu S, Sung E, Jang Y (2007). Higher concentrations of alanine aminotransferase within the reference interval predict nonalcoholic fatty liver disease. *Clinical Chemistry*.

[13] O'Brien RM, Granner DK (1991). Regulation of gene expression by insulin. *Biochemical Journal*.

[14] Nemesanszky E, Lott JA (1985). Gamma-glutamyltransferase and its isoenzymes: progress and problems. *Clinical Chemistry*.

[15] Karp DR, Shimooku K, Lipsky PE (2001). Expression of *γ*-glutamyl transpeptidase protects ramos B cells from oxidation-induced cell death. *The Journal of Biological Chemistry*.

[16] Hanigan MH, Ricketts WA (1993). Extracellular glutathione is a source of cysteine for cells that express *γ*-glutamyl transpeptidase. *Biochemistry*.

[17] The Korean Pediatric Society Health Statistics Committee (1999). *Standard Data Regarding Physical Development of Korean Children and Teenages*.

[18] Cho BK, Kang JH, Lee JS, Yu BY (2007). The usefulness of inbody 720 and anthropometric measurement compared with dual-energy x-ray absorptiometry as a diagnostic tool of childhood obesity. *Journal of the Korean Academy of Family Medicine*.

[19] Copieters Y, Parent F (2001). Blood pressure measurement in epidemiological investigation in teenagers. *European Journal of Epidemiology*.

[20] Matthews DR, Hosker JP, Rudenski AS, Naylor BA, Treacher DF, Turner RC (1985). Homeostasis model assessment: insulin resistance and *β*-cell function from fasting plasma glucose and insulin concentrations in man. *Diabetologia*.

[21] Ortega E, Koska J, Salbe AD, Tataranni PA, Bunt JC (2006). Serum *γ*-glutamyl transpeptidase is a determinant of insulin resistance independently of adiposity in Pima Indian children. *Journal of Clinical Endocrinology and Metabolism*.

[22] Knowler WC, Bennett PH, Hammam RF, Miller M (1979). Diabetes incidence and prevalence in Pima Indians: a 19-fold greater incidence than in Rochester, Minnesota. *American Journal of Epidemiology*.

[23] Baier LJ, Hanson RL (2004). Genetic studies of the etiology of type 2 diabetes in Pima Indians: hunting for pieces to a complicated puzzle. *Diabetes*.

[24] Goran MI, Gower BA (2001). Longitudinal study on pubertal insulin resistance. *Diabetes*.

[25] Whitfield JB (2001). Gamma-glutamyltransferase. *Critical Reviews in Clinical Laboratory Sciences*.

[26] Nilssen O, Forde OH, Brenn T (1990). The Tromso study: distribution and population determinants of gamma-glutamyltransferase. *American Journal of Epidemiology*.

[27] Paolicchi A, Emdin M, Ghliozeni E (2004). Human atherosclerotic plaques contain gamma-glutamyl transpeptidase enzyme activity. *Circulation*.

[28] Lee DH, Jacobs DR (2005). Association between serum gamma-glutamyltransferase and C-reactive protein. *Atherosclerosis*.

[29] Yamada J, Tomiyama H, Yambe M (2006). Elevated serum levels of alanine aminotransferase and gamma glutamyltransferase are markers of inflammation and oxidative stress independent of the metabolic syndrome. *Atherosclerosis*.

[30] Michael MD, Kulkarni RN, Postic C (2000). Loss of insulin signaling in hepatocytes leads to severe insulin resistance and progressive hepatic dysfunction. *Molecular Cell*.

[31] Schwimmer JB, Deutsch R, Rauch JB, Behling C, Newbury R, Lavine JE (2003). Obesity, insulin resistance, and other clinicopathological correlates of pediatric nonalcoholic fatty liver disease. *Journal of Pediatrics*.

[32] Marchesini G, Brizi M, Blanchi G (2001). Nonalcoholic fatty liver disease: a feature of the metabolic syndrome. *Diabetes*.

[33] Patel DA, Srinivasan SR, Xu JH, Chen W, Berenson GS (2007). Persistent elevation of liver function enzymes within the reference range is associated with increased cardiovascular risk in young adults: the Bogalusa Heart study. *Metabolism*.

[34] Kang YH, Min HK, Son SM, Kim IJ, Kim YK (2007). The association of serum gamma glutamyltransferase with components of the metabolic syndrome in the Korean adults. *Diabetes Research and Clinical Practice*.

[35] Sinaiko AR, Steinberger J, Moran A (2005). Relation of body mass index and insulin resistance to cardiovascular risk factors, inflammatory factors, and oxidative stress during adolescence. *Circulation*.

[36] Steinberger J, Moorehead C, Katch V, Rocchini AP (1995). Relationship between insulin resistance and abnormal lipid profile in obese adolescents. *Journal of Pediatrics*.

